# Substitutions in the Glycogenin-1 Gene Are Associated with the Evolution of Endothermy in Sharks and Tunas

**DOI:** 10.1093/gbe/evw211

**Published:** 2016-09-10

**Authors:** Adam G. Ciezarek, Luke T. Dunning, Catherine S. Jones, Leslie R. Noble, Emily Humble, Sergio S. Stefanni, Vincent Savolainen

**Affiliations:** ^1^ Department of Life Sciences, Imperial College London, Silwood Park Campus, Buckhurst Road, Ascot, UK; ^2^ Present address: Department of Animal and Plant Sciences, University of Sheffield, Sheffield, UK; ^3^ Institute of Biological and Environmental Sciences, School of Biological Sciences, University of Aberdeen, Zoology Building, Tillydrone Avenue, Aberdeen, Scotland, UK; ^4^ Present address: Department of Animal Behaviour, University of Bielefeld, Postfach 100131, Bielefeld, Germany; ^5^ Villa Comunale, Stazione Zoologica Anton Dohrn, Naples, Italy

**Keywords:** tuna, sharks, endothermy, positive selection, phylogenetics

## Abstract

Despite 400–450 million years of independent evolution, a strong phenotypic convergence has occurred between two groups of fish: tunas and lamnid sharks. This convergence is characterized by centralization of red muscle, a distinctive swimming style (stiffened body powered through tail movements) and elevated body temperature (endothermy). Furthermore, both groups demonstrate elevated white muscle metabolic capacities. All these traits are unusual in fish and more likely evolved to support their fast-swimming, pelagic, predatory behavior. Here, we tested the hypothesis that their convergent evolution was driven by selection on a set of metabolic genes. We sequenced white muscle transcriptomes of six tuna, one mackerel, and three shark species, and supplemented this data set with previously published RNA-seq data. Using 26 species in total (including 7,032 tuna genes plus 1,719 shark genes), we constructed phylogenetic trees and carried out maximum-likelihood analyses of gene selection. We inferred several genes relating to metabolism to be under selection. We also found that the same one gene, glycogenin-1, evolved under positive selection independently in tunas and lamnid sharks, providing evidence of convergent selective pressures at gene level possibly underlying shared physiology.

## Introduction

Bony fishes and sharks have been separated by up to 450 million years of independent evolution (Venkatesh et al. 2014). As such they differ in many aspects of their physiology, anatomy, and biochemistry (Bernal et al. 2001). Despite this, there is remarkable phenotypic convergence between two groups of active, epipelagic predators: the lamnid sharks (= family Lamnidae) and the tunas (= genera *Thunnus*, *Euthynnus*, *Auxis*, and *Katsuwonus* within family Scombridae). These two groups have a distinctive positioning of red muscle (RM), a specialized swimming style and can warm up regions of body (endothermy).

First, RM is used for slow-twitch contraction, as in steady state swimming. It is primarily fuelled by aerobic metabolism, and as such is relatively rich in mitochondria and myoglobin compared to white muscle (WM). This WM is fast-twitch muscle used for burst swimming, primarily fuelled by glycolysis (anaeroby). As such, WM has lower concentrations of myoglobin and mitochondria (Dickson 1996). Typically, in fish, the majority of RM is located superficially, close to the outside of the body. In contrast, in tunas and lamnid sharks, the majority of RM is located in a more central position within the body (Block and Finnerty 1994; Bernal et al. 2001). The centralization of RM has been directly associated with “thunniform” swimming and regional endothermy.

Second, “thunniform” swimming is characterized by the restriction of lateral movements to the caudal region (Donley et al. 2004; Gemballa et al. 2006). Force generated by the RM is transmitted efficiently to the tail, without causing local bending of a stiffened body (Westneat et al. 1993; Syme and Shadwick 2011). The RM is also a major source of metabolically generated heat. The evolution of regional endothermy requires a mechanism of insulation. In both groups, this is achieved using counter-current heat exchangers, which surround the centralized RM, enabling the maintenance of an elevated body temperature (Block and Finnerty 1994).

Third, endothermy is generally associated with high metabolic rates and high aerobic capacities (Nespolo et al. 2011). However, measuring metabolic rates in large, active fish is challenging (Blank et al. 2007). It is frequently stated that tunas have high mass-specific standard and maximum metabolic rates compared to ectothermic fish (Dickson and Graham 2004; Qiu et al. 2014). Measures of shark metabolic rates are mostly lacking, although high values have been recorded in the endothermic mako shark, *Isurus oxyrinchus* (Bernal et al. 2012). Measurements of the activity of metabolic enzymes have indicated that tuna’s WM have not only elevated anaerobic capacity, but also aerobic capacity, compared to ectothermic Scombridae (Dickson 1996). The same pattern has also been demonstrated in lamnid sharks (Bernal, Smith, et al. 2003).The elevated aerobic capacity of WM may enable rapid repayment of the oxygen debt induced by burst swimming, increasing speed of recovery (Korsmeyer and Dewar 2001; Bernal and Sepulveda 2005).

The genetic mechanisms underlying phenotypic convergence between laminid sharks and tunas are unknown. Although metabolic pathways are highly conserved across eukaryotes (Fernie et al. 2004), positive selection has been detected in enzymes of taxa under strong selective pressure for metabolic performance, such as consumption of very large prey in snakes or cold adaptation in insects (Castoe et al. 2008; Dunning et al. 2013). Lamnid sharks and tunas are also under strong pressure. Thunniform swimming and regional endothermy are associated with a high metabolic cost (Watanabe et al. 2015). This is particularly problematic due to the nutrient-poor pelagic environment these fish occupy (Korsmeyer et al. 1996) and the high rate of thermal diffusion in water, making heat retention difficult (Carey et al. 1971). These traits enable an increased thermal range (Dickson and Graham 2004) and cruise swimming speed (Watanabe et al. 2015) in RM endotherms and could therefore provide a strong selective benefit under conditions where this enables better access to high-energy prey (Madigan et al. 2015).

Phylogenetic approaches for detecting evidence of positive selection can be used to nominate candidate genes. Therefore here, we test two hypotheses: (i) given the elevated metabolic capacities of WM in endotherms, genes associated with muscle metabolism in lamnid sharks and tunas would have evolved under positive selection; and (ii) given convergent evolution between tunas and lamnid sharks, we expect to find orthologous genes involved in muscle metabolism to be under positive selection in both groups. To test these hypotheses, we sampled the WM of a range of endothermic tunas and sharks along with their closest ectothermic relatives. We sequenced their WM transcriptomes, which we also supplemented with published RNA-seq data. We then applied comparative phylogenetic analyses search for candidate genes for selection, which may underlie the phenotypic convergences observed in lamnid sharks and tunas.

## Materials and Methods

### Sampling

WM samples of two lamnid sharks and one ectothermic species (basking shark; table 1), and seven Scombridae species, including six endothermic tunas, and ectothermic mackerel were collected between January 2013 and February 2014. This data was supplemented by previously published RNA-seq data for 11 species (table 1). All samples were stored at −20 °C in RNALater (Sigma-Aldrich, St. Louis, MO). Prior to RNA extraction, all samples were disrupted and homogenized using the Powergen homogeniser (Fisher Scientific, Loughborough, UK). Total RNA was extracted and purified using the RNeasy Fibrous Tissue Mini Kit and MiniElute Cleanup Kit (Qiagen, Venlo, Netherlands) following the manufacturer’s protocol. RNA quality and quantity were assessed using a Nanodrop ND2000 (Nanodrop Technologies, Wilimgton, DE), a TAE-agarose gel and an Agilent 2100 Bioanalyzer (Agilent Technologies, Palo Alto, CA).

**Table 1:** The origin of samples used for this study and *de novo* trinity assembly statistics

Common name	Species name	Origin	Paired-end reads used for assembly (million)	Number of assembled contigs	Contig N50	Number of coding regions after clustering
Yellowfin tuna	*Thunnus albacares*	Purchased, UK	57.9	61,045	1,851	18,343
Atlantic bluefin tuna	*Thunnus thynnus*	Purchased, UK	58	76,764	1,593	21,922
Bigeye tuna	*Thunnus obesus*	Purchased, UK	59.9	74,882	1,851	21,066
Skipjack tuna	*Katsuwonus pelamis*	Azores	59.3	83,724	1,414	20,385
Southern bluefin tuna	*Thunnus maccoyii*	Australia	53.9	58,944	1,017	15,170
Albacore tuna	*Thunnus alalunga*	Australia	53.8	81,372	1,967	22,263
Atlantic mackerel	*Scomber scombrus*	Purchased, UK	58.2	65,763	761	15,335
Atlantic bonito	*Sarda sarda*	Assembly kindly provided by the authors of Sarropoulou et al. (2014)	162.1	68,220	3,011	27,010
Pacific bluefin tuna	*Thunnus orientalis*	Supplementary information of Yasuike et al. (2016)	–	40,813	1,722	28,471
Black scabbardfish	*Aphanopus carbo*	Stefanni et al. (2014)	–	8,319	619	1,055
Yellowtail kingfish	*Seriola lalandi*	SRR2138320	95.7	138,558	2,204	34,218
Barramundi	*Lates calcarifer*	GAQL01000001.1-01363785.1	–	363,785	1,680	54,776
Porbeagle	*Lamna nasus*	UK	16.6	53,103	708	8,694
Shortfin mako shark	*Isurus oxyrinchus*	Azores	81.4	81,680	892	15,046
Great White shark	*Carcharadon carcharias*	ORFs taken from Richards et al. (2013)	–	105,313	640	17,134
Sandtiger shark	*Carcharias taurus*	SAMN03333352	71.7	118,363	1,687	24,769
Basking shark	*Cetorhinus maximus*	UK	61.5	19,017	343	1,630
Smooth dogfish	*Mustelus canis*	SAMN03333350	52.7	98,463	2,026	19,990
Lemon shark	*Negaprion brevirostris*	SAMN03333351	62.3	70,506	1,701	16.217
Caribbean reef shark	*Carcharhinus perezii*	SAMN03333349	62	111,848	2,340	23,075
Bull shark	*Carcharhinus leucas*	SAMN03333348	60.5	91,122	1,719	21,657
Blue shark	*Prionace glauca*	SAMN03333347	65.8	96,740	1,137	17,669
Tiger shark	*Galeocerdo cuvier*	SAMN03333353	59.1	179,867	1,858	26,843
Atlantic sharpness shark	*Rhizoprionodon terraenovae*	SAMN03333345	60.5	88,870	1,844	19,646
Small-spotted catshark	*Scyliorhinus canicula*	http://skatebase.org; last accessed March 2014	–	107,231	695	24,218
Blacknose shark	*Carcharhinus acronotus*	SAMN03333346	57.8	131,575	2,201	22,956

In order to verify species identity of fish purchased from traders, we sequenced cytochrome *b* (*cytB*) (Botti and Giuffra 2010). A cDNA reverse transcription kit (Applied Biosciences Inc, Foster City, CA) was used to generate cDNA following the manufacturers protocol. PCR amplifications were carried out using a RedTAQ ReadyMix PCR Reaction Mix (Sigma-Aldritch, St. Louis MO) using primers adapted from Botti and Giuffra (2010) (supplementary table S1, Supplementary Material online) and a Veriti Thermal Cycler (Applied Biosciences Ltd, Foster City CA). PCR products were then purified with ExoSAP-IT (Affymetrix Inc, Santa Clara, CA) and sequenced using Big Dye Terminator v3 (Applied Biosciences Inc, Foster City, CA). Sequencing product was subsequently cleaned using ethanol and sodium acetate precipitation, and run on a 3130xl Genetic Analyzer (Applied Biosciences Inc, Foster City CA). Electropherograms were edited using Geneious (v6) and blastn-searched against GenBank.

### Construction and Sequencing of cDNA Libraries

We commissioned 3′-fragment normalized cDNA libraries for construction by an external company (BGI Tech Solutions, Hong Kong). Using The TruSeq RNA Library Preparartion Kit v2, cDNA libraries were produced with DSN normalization. These normalized cDNA libraries were then sequenced using Illumina HiSeq 2000 (Illumina Inc, San Diego, CA). Initial quality control was carried out by BGI Tech Solutions, with low quality reads (phred score <20) removed and primer and adaptor sequences trimmed. Upon retrieval, cleaned reads were evaluated using FastQC (v0.10.1), and then assembled into contigs using Trinity (v2013-08-05) (Grabherr et al. 2011), with default settings. Data are available on GenBank under Bioproject number PRJNA305977.

### Gene Prediction and Annotation

For each transcript, the longest open reading frame (ORF) was extracted using TransDecoder [trinity package: (Grabherr et al. 2011)]. Stop codons as well contigs that returned more than one TransDecoder ORF were removed from the dataset. To reduce redundancy, each set of ORFs was clustered using CD-HIT-EST using a cut-off of 0.98 (Fu et al. 2012). A phylogenetic tree-based approach was then used to detect orthologs between the sharks and between the perciform fish separately. Clustered ORF assemblies were translated. To guide orthogroup assignment, cDNA sequences for *Danio rerio*, *Homo sapiens*, *Mus musculus*, *Latimeria chulmnae*, and *Oryzias latipes* were downloaded from the Ensembl database (Yates et al. 2015). Using TransDecoder, coding sequences were extracted from each contig. These contigs were then clustered using CD-HIT-EST using a cut-off of 0.98, and translated. Orthofinder was used to infer homolog groups (Emms and Kelly 2015). The first step of this was an all-versus-all blastp (v2.2.25) search (Altschul et al. 1990). Orthofinder then normalizes blast scores for sequence length and phylogenetic distance before selecting putative gene pairs for orthogroup inference using the MCL clustering algorithm (Van Dongen 2000). Following the methods and scripts of Yang and Smith (2014), we then trimmed orthogroup trees. First, terminal branches with an absolute length of 2, or relative length of 10 times that of their sister were trimmed. As RNA-seq data includes multiple splice variants and isoforms, monophyletic or paraphyletic groups can arise from the same species. In these instances, the contig with the highest number of aligned characters was retained, with the remainders trimmed. Deep paralogs, with a branch length of greater than 0.5, were then cut. A raw coding sequence file was then generated for each orthogroup. This was re-aligned using mafft (v7.2.45) (Katoh and Standley 2013), and phylogenetic trees inferred using RAxML (v8.1.17) (Stamatakis 2006). Following a repeat of the trimming procedure, orthologs were inferred using the “prune_paralogs_RT.py” script of Y. Yang and Smith (2014). This RT method explicitly accounts for gene duplications. *Danio rerio*, *H. sapiens*, *M. musculus*, *L. chulmnae*, and *O. latipes* were used here as outgroups to root the trees, but then were trimmed. For downstream analyses, we only looked at putative orthologs that were identified across at least five species and included at least two endothermic and two ectothermic species.

Alignment error has been demonstrated to be a key source of false positives in positive selection inferences (Markova-Raina and Petrov 2011; Redelings 2014). To reduce the likelihood of this, we used a stringent alignment approach. The putatively orthologous nucleotide sequences were first translated to proteins. Using m-coffee, implemented within the tcoffee v11 software package (Notredame et al. 2000), these amino acid sequences were aligned using four separate aligners: muscle_msa, mafftgins_msa, tcoffee_msa and kalign_msa. Output scores were given for each alignment site based on the concordance of the different aligners. All sites with a concordance less than nine, which indicates total concordance, were trimmed. Regions in the resultant alignments that are highly divergent may not be truly orthologous, or still may be influenced by alignment, sequencing or assembly error. To further control for this, alignment quality of each column was analyzed using the Transitive Consistency Score (TCS) alignment evaluation score implemented within t-coffee (Chang et al. 2014). Only columns with the maximum quality score of nine were retained. These trimmed protein sequences were back-translated to their corresponding nucleotides. Codons absent in at least half of the species were removed. Maxalign v1.1 (Gouveia-Oliveira et al. 2007) was then used to detect and remove poorly aligned gap-rich sequences. This reduces the risk of paralogous sequences being analyzed, as these are less likely to align well. All sequences which had sequences discarded by maxalign were then checked to see if the remaining good quality aligned sequences still considered enough species to be considered (using the same criteria outlined earlier). If this was the case, they were realigned and trimmed, with the low quality sequences removed, using the same method and put forward for analysis.

All trinity transcripts and ORFs corresponding to good quality alignments were annotated using the Trinity Trinotate pipeline (Grabherr et al. 2011). Sequences were searched against UniProt using the SwissProt and Uniref90 databases (E-value cutoff 1E−10). Coding sequences were also searched for conserved protein domains using Pfam (Finn et al. 2014). Additionally, ORFs were BLASTx-searched against NCBIs nr database and annotated using Blast2go v2.5 (Conesa et al. 2005).

### Phylogenetic Inferences

Analyses for positive selection using the PAML software require an accurate phylogenetic tree (Yang 2007). Separate phylogenetic trees were inferred for the sharks and bony fish (including tunas). In each case, 4-fold degenerate sites were extracted from each putative ortholog, and concatenated to produce a 4-fold supermatrix. This ensures that phylogeny reconstruction was independent from detecting positive selection because 4-fold degenerate sites do not affect the sequence of amino acids in the translated protein. Using phyutility (v2.2.26), alignment columns with less than half of species present were trimmed. A maximum-likelihood phylogenetic tree was built for each dataset, using RAxML (v8.1.18), with 1,000 rapid bootstraps (Stamatakis 2006; Stamatakis et al. 2008). In each case, the model of evolution was determined using the best Akaike information criteria (AIC) scores (Posada and Buckley 2004), using jModeltest v2.1.10 (Posada 2008). For each dataset, a Bayesian phylogenetic tree was also inferred using ExaBayes (Aberer et al. 2014). Four independent MCMC runs, each with three coupled chains, were run for 1,000,000 generations, sampling every 500. Using the “sdsf” and “postProcParam” tools of exabayes, along with Tracer (v1.6) (Rambaut et al. 2013), we ensured that average deviation of split frequencies was close to zero, potential scale reduction factors were close to one and effective sample sizes of estimated parameters were greater than 200.

### Detecting Positive Selection

The CodeML programme of the PAML (v4.7) package was used to analyze all alignments for positive selection (Yang and Bielawski 2000). The branch-site test was implemented for each ortholog (Zhang et al. 2005). These models require the specification of a “foreground” branch, which can be tested for evidence of selection. As our hypothesis relates to selection underlying the evolution of endothermy, the root branch of the endothermic taxa was selected and subjected to a branch-site test implementing two models. One model allowed for selection with four site classes: 0 *< ω <* 1 in both branch-classes, *ω* = 1 in both, *ω*foreground* >* 1=*ω*background and *ω*foreground *>* 1*>ω*background, in which *ω* denotes the ratio of the number of non-synonymous substitutions per non-synonymous site (d_N_) to the number of synonymous substitutions per synonymous site (d_S_). The null model differs in that *ω*foreground cannot exceed 1. A likelihood ratio test (LRT) with *χ*1^2^ was then used to compare models, and test whether the model allowing for selection fits the data significantly better than the null model. As different genes contained different numbers of species, a newick-formatted tree file was generated for each individual gene. When taxa were absent, they were removed from a base tree with all taxa present, using Newick Utilities (Junier and Zdobnov 2010). Three runs with different starting *ω* values (0.5, 1, 1.5) for each gene were carried out for each alignment.

A key source of false-positive in the branch-site model is alignment error (Fletcher and Yang 2010; Markova-Raina and Petrov 2011), which even with strict alignment procedures cannot be conclusively eliminated. All genes with LRT *P <* 0.05 were considered putatively under selection and independently re-analyzed using a different alignment procedure. These orthologs were translated and aligned using PRANK v100802 (Löytynoja and Goldman 2005) implemented through Guidance v1.5 (Penn et al. 2010). PRANK has been demonstrated to produce a low rate of false-positives in branch-site tests compared to other alignment softwares (Markova-Raina and Petrov 2011; Redelings 2014). Guidance evaluates the quality of alignments using two methods: the heads or tails method, which evaluates uncertainty generated by co-optimal solutions and a Guidance method, which analyses alignment robustness to guide-tree uncertainty. These raw alignments were then back-translated to nucleotides. Protein alignment residues with a low score (using the default recommendation of ≤0.93) were removed. Alignments were then parsed to Trimal v1.3 (Capella-Gutiérrez et al. 2009) where columns with gaps in at least 40% of sequences or with a similarity score of *<*0.001 were trimmed. These trimmed protein alignments were then used to trim the nucleotide alignment. As with the original alignments, these were assessed using Maxalign and realigned without excluded taxa if necessary. These alignments were then analyzed with the branch-site test implemented through a different software, slimcodeml (*v*2013 − 02 − 07). As with the t-coffee alignments, each gene was analyzed with the same three different starting *ω* values. For each ortholog, all *P* values generated by separate branch-site tests (i.e., between the different alignment methods and starting *ω*) were corrected using the method of Benjamini–Hochberg (Benjamini and Hochberg 1995). We only considered genes to be under selection if *P* values were *<*0.05 (Benjamini–Hochberg adjusted for all tests on the same gene) for all six tests carried out. Genes found to be under-selection were tested for enrichment, using all the genes analyzed as the background. A Fisher's exact test, with Benjamini–Hochberg adjustment for multiple comparisons, was implemented in Blast2go v2.8.0 (Conesa et al. 2005), based on the gene ontology (GO) annotations from the nr database. They were also analyzed using the CodeML free-ratio and one-ratio tests. Genes with an overall d_S_ > 1 in the one-ratio test, or d_S_ > 1 in the endothermic root in the free-ratio test, were inferred to be influenced by synonymous-site saturation. This may influence the reliability of the branch-site test (Gharib and Robinson-Rechavi 2013; Roux et al. 2014).

Genes with the same gene annotation found to be under selection in both the lamnid sharks and tunas were investigated further. To assess whether these genes have a particularly overall high rate of d_N_ or d_N_/d_S_, free-ratio and one-ratio tests were carried out in CodeML. High rates of non-synonymous mutations may make a gene a more likely target of positive selection, as there is an increased chance of an advantageous allele arising (Montoya-Burgos 2011). To assess whether orthology inference was accurate, coding sequences from genome projects on the corresponding gene tree were downloaded from Ensembl (Yates et al. 2015). These were aligned with the corresponding orthologs from our dataset using mafft (v7.245). The gene tree was then constructed using RAxML with 200 rapid bootstraps (v8.1.17).

### Ancestral State Reconstructions

For genes inferred to be under selection in both tunas and lamnid sharks, we used ancestral state reconstructions, inferring specific amino acid substitutions using FastML (v3.1) (Ashkenazy et al. 2012). We then compared the ancestral amino acid sequence of the endotherms to the ancestral sequences of their closest ectotherms. We visualized these changes on a human high-resolution crystal structures (Chaikuad et al. 2011), downloaded from the Protein Data Bank in Europe (Velankar et al. 2010).

## Results and Discussion

### Orthologous Genes

Between 16.6 and 81.4 million paired-end reads were sequenced for each species (mean 58 million; table 1). Using tree-based orthology detection techniques, an initial 12,982 bony fish and 6,620 shark orthologs were detected. Following the t-coffee alignment pipeline, 7,798 and 2,086 bony fish and shark orthologs remained for phylogenetic analyses. To detect positive selection using PAML, we needed orthologs present in the sisters to tunas and lamnid sharks (namely, Atlantic bonito and sandtiger shark), which comes down to 7,032 bony fish and 1,719 shark genes (supplementary table S2, Supplementary Material online).

### Phylogenetic Trees

We built two supermatrices consisting of all of the 4-fold degenerate sites from the 7,798 and 2,086 orthologs above. This resulted in a matrix of 701,592 nucleotides for bony fish and 173,967 for sharks. The “GTRGAMMA” model of evolution was used in each maximum-likelihood inference. The percentage of gaps for each species ranged from 10.1% to 97% in bony fish and 8.2% to 89.7% in sharks (supplementary table S3, Supplementary Material online).

**Fig. 1.—:**
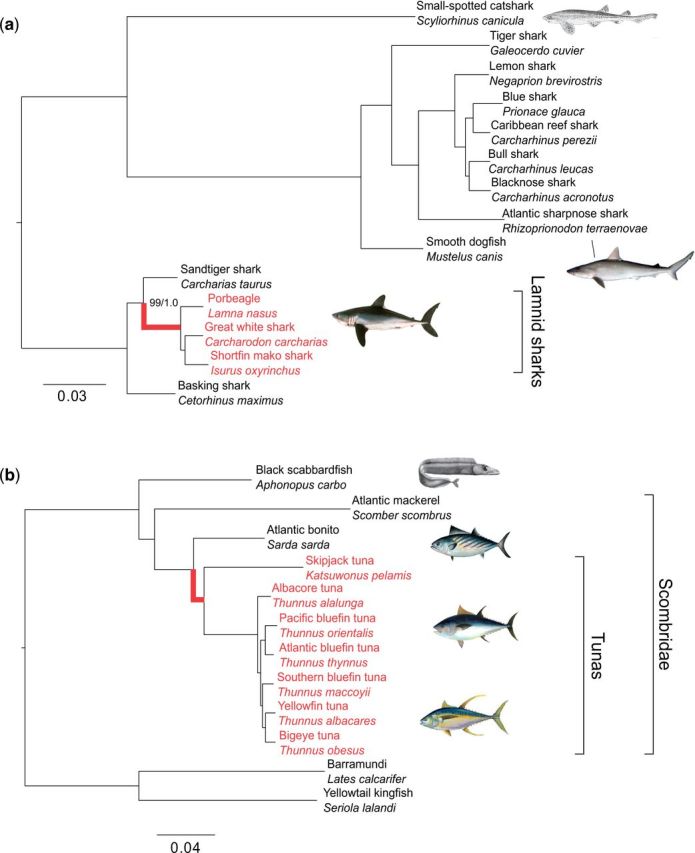
Phylogenetic tree of sharks (*a*) and bony fish (*b*). Endothermic species as well as their root branch are in red. All nodes were fully supported (1.0 posterior probability, 100% bootstrap support) unless otherwise indicated. Scale bar refers to branch length (number of expected substitutions per site). Images taken from http://en.wikipedia.org, http://commons.wikipedia.org.

We recovered a strongly supported phylogenetic tree for sharks, with all branches receiving 99–100% bootstrap and posterior probability of 1.0 (fig. 1a). This agreed with recent studies that used five genes (Vélez-Zuazo and Agnarsson 2011), but not with Sorenson et al. (2014) in placing *Carcharias taurus* as the closest relative to the endothermic sharks (rather than *Cetorhinus maximus*). Notably, data of *C. maximus* were available only in 10.3% of our matrix. The sample came from an individual stranded dead on a beach, which may have reduced RNA quality. Only 1,630 clustered, filtered coding sequences were analyzed for this species, compared to a mean of 18,593 for the other sharks. However, all nodes received bootstrap support 100% and posterior probability of 1.0, indicating that there was still sufficient data for *C. maximus*.

The bony fish dataset also produced a strongly supported tree (fig. 1b). Although *Aphanopus carbo* was poorly represented in the matrix (data present only for 3.0% of the aligned sites), it was confidently placed in a position consistent with previously published trees (Miya et al. 2013). Relationships amongst the *Thunnus* species were in agreement with a recently published RAD-seq based phylogenetic tree (Díaz-Arce et al. 2016).

### Positive Selection

After the two alignment procedures (see “Methods” section), 139 genes (1.9%) were inferred to be under positive selection in tunas, and 19 (1.1%) in the lamnid sharks (supplementary table S4, Supplementary Material online). No evidence for GO term enrichment was found in either dataset. We found evidence of synonymous-site saturation in three genes inferred to be under selection in the lamnid sharks (saturation inferred from an overall d_S_ > 1*: MYG*: 1.14*, BTNL1*: 1.34 and an unidentified protein: 3.7897). However, there was no significant evidence of enrichment of genes inferred to be under selection with overall d_S_ > 1 compared to all genes tested (Fisher’s exact test, *P* = 0.28). We found evidence of saturation in ten of the tuna genes (overall d_S_ > 1: *AATC*: 1.08, *TNF6CB*: 1.50, *RIR1*: 1.00, *LYG:* 1.00, *IRF8:* 1.00, *CD37:* 1.21, *COPT1*: 1.20, *PBDC1:* 1.09, *RN214:* 1.34, *MYSM1*: 1.04). As with sharks, there was no significant enrichment (Fisher's exact test, *P* = 0.64) indicating that it was not a significant cause of false positives. Indeed, simulation studies have suggested that a high d_S_ causes a lack of power in the branch-site test rather than an excess of false-positives (Gharib and Robinson-Rechavi 2013).

One gene was inferred to be under selection in both tunas and lamnid sharks independently: the glycogenin-*1* gene (*GLYG1*) (supplementary table S4, Supplementary Material online). This is unlikely due to chance (we estimated the probability of a given gene been under selection in both groups independently to be 0.0002, given that 1,192 genes with the same gene name were present in both groups, of which 14 were under selection in sharks and 22 in tunas). This crude estimation assumes that genes are equally likely to be under selection. To examine whether *GLYG1* is a particularly fast evolving gene, we performed free-ratio and one-ratio tests using CodeML, to test whether *ω* is high either overall or in background branches. In bony fish, overall *ω* for the *GLYG1* gene was 0.21, which was within one standard deviation (0.16) of the mean *ω* of all bony fish genes tested (0.14). The free-ratio model revealed an *ω* of 0.40 in the root of tunas and 0.83 at the root of lamnid sharks. All other branches with high values of *ω* (branches with 1.06, 1,18, and 0.79 within the tunas, one with 999 within the sharks) were supported by low values of d_S_ (<0.00001), rather than elevated d_N_, indicating low genetic differentiation rather than a fast-evolving gene. The overall *ω* was 0.13 in the shark *GLYG1* gene. This was also within one standard deviation (0.16) of the mean *ω* of all shark genes tested (0.17). These results indicate that *GLYG1* is not a particularly fast evolving gene in these taxa. However, sampling of this gene with greater phylogenetic coverage may provide further information as to whether it has undergone selection in separate groups.

Note that we did not have *GLYG1* data in the following taxa: *Katsuwonus pelamis*, *Thunnus alalunga*, *Thunnus obesus, A. carbo, Lates calcarifer, Seriola lalandi*, *Lamnua nasus*, *C. maximus*, and *Prionace glauca*.

To confirm that we were analyzing true orthologs of *GLYG1*, we inferred a gene tree using *GLYG1* genes from our data sets, along with *GLYG1*, paralogs *GLYG1a*, *GLYG1b*, and *GLYG2* genes from other genomes. All our bony fish genes clustered within the *GLYG1a* teleost genes (supplementary fig. S1, Supplementary Material online). Similarly, all our shark genes clustered together as sister to coelacanth and tetrapod *GLYG1* genes (supplementary fig. S1, Supplementary Material online). These results support our ortholog inference rather than comparing multiple isoforms. Although there has been a gene duplication in *GLYG1* in bony fish (Yates et al. 2015), the isoform we analyzed here is *GLYG1a*.

*GLYG1* is a candidate gene for recovery following the predatory behavior of tunas and lamnid sharks. The glycogenin encoded by *GLYG1* is an enzyme involved with the muscular genesis of glycogen, which is particularly important in fast-twitch muscle (Cussó et al. 2003). The rate at which muscular glycogen is restored following exercise dictates how quickly an individual can recover from exercise. It has been demonstrated that the tuna *K. pelamis* can do this rapidly, at a rate similar to mammals (Weber et al. 1986). In humans, increased expression of *GLYG1* has been found during recovery from exercise (Kraniou et al. 2000), and has been associated with increased muscular glycogen content (Zhang et al. 2013). Mutations in this gene also result in glycogen depletion of the skeletal muscle (Nilsson et al. 2012). Although measurements of muscular glycogenin genesis have not been made in lamnid sharks, high activities of both lactate dehydrogenase (LDH) and citrate synthase (CS) have been documented compared to their ectothermic relatives (Bernal, Smith, et al. 2003). These are markers of anaerobic and aerobic metabolic capacity, respectively (Dickson 1996). The relative abundance of these enzymes has also been found to positively correlate, and so it has been speculated that endothermic sharks are able to clear lactate in a similar manner to tunas (Bernal and Sepulveda 2005). This suggests a similarly elevated rate of exercise recovery.

**Fig. 2.—:**
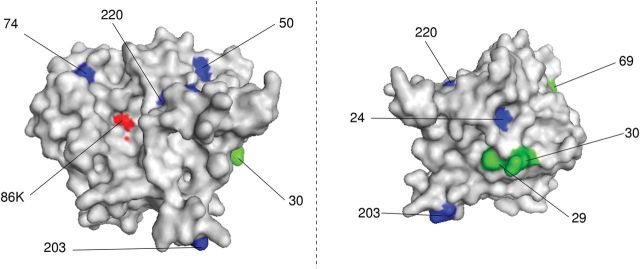
Two views of the structural modelling of human glycogenin-1, showing amino acids changes between endothermic and ectothermic fish (see text for details): changes endothermic sharks in blue, changes in endothermic tunas in green, active site in red Lysine 86; other numbers refer to amino acid position in the human protein.

We also inferred the ancestral sequences of *GLYG1* in our trees. Mapping amino acid changes on human *GLYG1* shows that these are at the surface of the protein, with the exception of one change in tunas (fig. 2). Such surface sites are likely to influence thermal stability of the enzyme (Fields 2001), although not excluding an effect on catalytic performance (Fields and Somero 1998).

Given the large genome size of sharks (Venkatesh et al. 2014) and evolutionary distance between sharks and tunas, it is not surprising that we did not find substitutions at convergent amino acid sites within *GLYG1*. Convergent evolution is expected to be more common in organisms with small genomes, as there are fewer mutational target sites which could influence fitness (Stern 2013). Additionally, the fitness effects of substitutions are dependent on the genetic background. The great evolutionary distance between tunas and sharks is likely to have reduced the probability of parallel or convergent substitutions at the same sites (Storz 2016).

We also found evidence of selection in other genes associated with metabolism (supplementary table S4, Supplementary Material online). This included one electron transport chain gene (*COX41*) in lamnid sharks. This gene was tested, but not inferred to be under selection in the tunas. Four lipid metabolism genes (*MCAT*, *ACOT1*, *ACOT4*, and *ACOT13*) were inferred to be under selection in tunas along with two genes associated with glycolysis (*TPISB, TIGRA*). Of these genes, only *ACOT4* was tested in sharks, and was not inferred to be under selection.

The electron transport chain gene inferred to be under selection in the sharks, *COX41*, encodes a subunit of cytochrome-*c.* This is the last enzyme in the electron transport chain, and plays a key role in aerobic respiration (Wikström 2010). In tunas, three *ACOT* (Acyl-CoA-synthetases) were inferred to be under selection. Acyl-CoA-synthetases facilitate β-oxidation, by providing CoA (Hunt et al. 1999). *MCAT* also may play a role in facilitating β-oxidation. Mitochondrial carnitine/acylcarnitine carrier proteins catalyse transport of acylcarnitine into the mitochondria, increasing fatty acyl units in the mitochondrial matrix, where β-oxidation enzymes oxidize them (Indiveri et al. 2011). β-oxidation is vital to overall production of metabolic energy, where fatty acids are broken down to form acetyl coenzyme A, which enters the tricarboxylic acid cycle and feeds aerobic respiration (Indiveri et al. 2011). The protein encoded by *GPDA* plays a variety of metabolic roles, linking lipid metabolism to gluconeogenesis (Harding et al. 1975) and contributing electrons to the mitochondrial electron transport chain (Bunoust et al. 2005). This indicates adaptive evolution relating to aerobic metabolism occurred in the evolution of tunas and lamind sharks, although not in the same pathways.

The protein encoded by *TPISB* operates at a branch-point influencing glycolytic flux (Compagno et al. 1999). As branch-point enzymes exhibit control over rate of glycolysis, such enzymes are likely to be a target of selection (Eanes 2011). *TIGRA* encodes a probable fructose-2,6-bisphsphatase. There is evidence that this controls phosphofrucokinase-*1* (a key glycolysis regulatory enzyme) and therefore the rate of glycolysis (Hue and Rider 1987). This indicates a selective pressure influencing glycolytic capacity in tunas, but not in sharks. Although higher than other sharks, the metabolic capacities of lamnid shark WM are still lower than those of the tunas (Bernal, Smith, et al. 2003). This indicates that such adaptive evolution did not occur in the lamnid sharks, and that different mechanisms underlie its elevated glycolytic potential.

A further six genes with functions relevant to the physiology and behavior of tunas were inferred to be under selection (i.e., *MPSF, MYOZ2, LMOD3, RYR1*, and *MOT4*). These have functions relating to muscular contraction, muscular development and transmembranal lactate transport (van der Ven and Fürst 1997; Franzini-Armstrong 1999; Yuen et al. 2014; The UniProt Consortium 2015). Orthologs for these genes were not tested in sharks, and genes with similar functions were not inferred to be under selection. However, a myoglobin gene, *MYG*, was. Higher levels of myoglobin have been documented in the red muscle of endothermic sharks than ectothermic sharks, which would enhance diffusion of oxygen from the blood to the muscle cells (Bernal, Sepulveda, et al. 2003). *MYG* was tested, but not inferred to be under selection in the tunas.

## Conclusion

We hypothesized that selection would have acted on genes involved in metabolic pathways, as well as genes relating to muscular contraction and development. We found several such genes, including *COX41, TPISB, TIGRA*, *MCAT*, *ACOT1*, *ACOT4*, *ACOT13, MPSF, MYOZ2, LMOD3, RYR1*, and *MOT4*. We also hypothesized that the same genes will be found to be involving under positive selection in both lamnid sharks and tunas. We found this to be the case only for one gene, *GLYG1*, which may have had a role in enhancing exercise recovery in the WM in each group. Further studies are needed to investigate how these amino acid substitutions are affecting the function of the enzyme.

Of course, the evolution of endothermy is more complex that involving just one gene. For example, ontogenetic studies in these fish should reveal the mechanisms underlying the centralization of RM, which is key to the phenotypic convergence in lamnid sharks and tunas. Further work, focusing on sequence evolution as well as gene expression in other tissues, such as RM, cardiac tissue, brain, and liver is needed. Our study was restricted to WM, in which not all of the individual’s genes were expressed, and therefore not sequenced here. We actually used in our final dataset only 1,719 and 7,032 genes in the sharks and tunas, respectively, representing a relatively small proportion of the estimated 20,000–25,000 genes that must be present in these fish (Braasch et al. 2016). Increasing phylogenetic coverage is also likely to provide valuable insights. For example, the thresher shark, *Alopias vulpinus* may have evolved RM centralization and endothermy independently to lamnid sharks, but does not share the “thunniform” swimming or enhanced white muscle metabolic capacity (Bernal, Smith, et al. 2003; Bernal and Sepulveda 2005). Similarly, the slender tuna, *Allothunnus fallai*, has centralized RM and regional endothermy, but its phylogenetic position is still unclear (Sepulveda et al. 2008; Santini et al. 2013); it was not sampled here.

There are few examples of gene convergence underlying the same trait in distantly related taxa. One famous example includes echolocation in bats and cetaceans, whose evolution was first reported to involve convergent changes in nearly 200 genes (Parker et al. 2013). It was subsequently documented that this did not exceed the background level of amino acid convergence between echolocating and non-echolocating lineages, even in hearing genes (Zou and Zhang 2015; Thomas and Hahn 2015). This exemplifies the difficulty to infer the genetic basis of complex traits. We hope we have contributed to elucidating some of the remarkable convergence between sharks and tunas, but we await further studies that look into a broader taxonomic coverage and detailed functional assays.

## Supplementary Material

Supplementary Data
