# Supplementary material for: Substitutions in the Glycogenin-1 Gene Are Associated with the Evolution of Endothermy in Sharks and Tunas
Source: Genome Biol Evol. 2016 Sep 10;8(9):3011–21. doi: 10.1093/gbe/evw211 (PMC5630876; doi:10.1093/gbe/evw211)
Supplement: Supplementary Data [file evw211_Supplementary_Data.zip › Supplementary.docx]

### Substitutions in the glycogenin-1 gene are associated with the evolution of endothermy in sharks and tunas – Supplementary information

### Adam G Ciezarek, Luke T Dunning, Catherine S Jones, Leslie R Noble, Emily Humble, Sergio Stefanni, Vincent Savolainen

Supplementary Figures


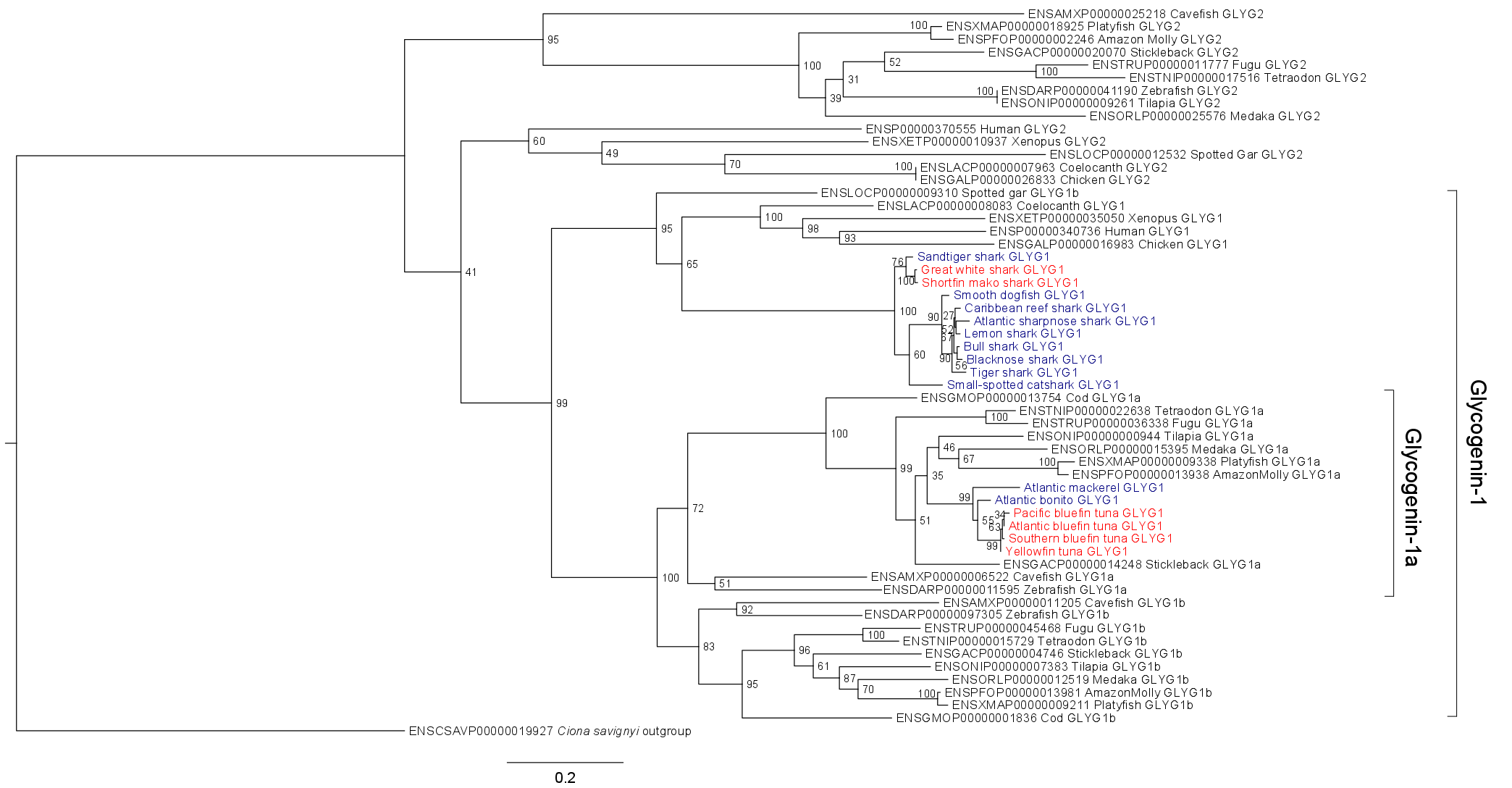


Figure S1. Phylogenetic reconstruction of *GLYG1*, *GLY1a*, *GLYG1b* and *GLYG2* genes from our dataset and the Ensembl database. Ensembl protein IDs are given in the tip labels, along with common name of the species and its isoform. Species highlighted in red are endothermic species from our dataset; those in blue ectothermic species from our dataset.

Supplementary tables

Table S1: Primers used for cytochrome *b* amplification for scombrid species verification

| Primer name | Primer sequence 5’-3’ |
| --- | --- |
| cytochrome-*b* Forward | AACGGGGCCTCTTTCTTCTT |
| cytochrome-*b* Reverse | GTGGCGTTGTCTACTGAAAAGCC |

Table S2: Missing taxa from filtered orthologous datasets used for PAML analysis.

| Scombrid orthologs | | Shark orthologs | |
| --- | --- | --- | --- |
| Total ortholog number | 7,032 | Total ortholog number | 1,719 |
| Number of species | Number of orthologs | Number of species | Number of orthologs |
| 5 | 1,334 | 5 | 66 |
| 6 | 1,180 | 6 | 49 |
| 7 | 1,161 | 7 | 66 |
| 8 | 1,098 | 8 | 84 |
| 9 | 1,037 | 9 | 147 |
| 10 | 738 | 10 | 209 |
| 11 | 413 | 11 | 408 |
| 12 | 71 | 12 | 409 |
|  |  | 13 | 258 |
|  |  | 14 | 23 |
| Species | Number of orthologs where present | Species | Number of orthologs where present |
| *S. scombrus* | 1,901 | *M. canis* | 1,335 |
| *S. sarda* | 7,032 | *N. brevirostris* | 1,325 |
| *K. pelamis* | 4,392 | *C. perezii* | 1,455 |
| *T. alalunga* | 5,535 | *C. leucas* | 1,376 |
| *T. thynnus* | 4,426 | *C. acronotus* | 1,464 |
| *T. maccoyii* | 2,652 | *C. maximus* | 240 |
| *T. obesus* | 5,096 | *C. taurus* | 1,719 |
| *T. albacares* | 4,866 | *L. nasus* | 1,336 |
| *T. orientalis* | 4,544 | *I. oxyrinchus* | 1,544 |
| *A. carbo* | 265 | *C. carcharias* | 1,215 |
| *L. calcarifer* | 6,087 | *P. glauca* | 1,405 |
| *S. lalandi* | 5,973 | *R. terraenovae* | 1,401 |
|  |  | *S. canicula* | 1,017 |
|  |  | *G. cuvier* | 1,425 |

Table S3: Sites present, and percentage of sites absent for the corresponding 4-fold degenerate supermatrix for each species present in the study.

|  | Present | Total | Present% | Absent% |
| --- | --- | --- | --- | --- |
| *T. thynnus* | 560527 | 701592 | 79.89358 | 20.10642 |
| *T. orientalis* | 487321 | 701592 | 69.45932 | 30.54068 |
| *T. maccoyii* | 358172 | 701592 | 51.05132 | 48.94868 |
| *T. obesus* | 616,706 | 701592 | 87.90095 | 12.09905 |
| *T. albacares* | 599120 | 701592 | 85.39436 | 14.60564 |
| *T. alalunga* | 626418 | 701592 | 89.28523 | 10.71477 |
| *K. pelamis* | 523719 | 701592 | 74.64723 | 25.35277 |
| *S. sarda* | 563801 | 701592 | 80.36024 | 19.63976 |
| *S. scombrus* | 197830 | 701592 | 28.1973 | 71.8027 |
| *A. carbo* | 21337 | 701592 | 3.041226 | 96.95877 |
| *S. lalandi* | 621979 | 701592 | 88.65252 | 11.34748 |
| *L. calcarifer* | 630558 | 701592 | 89.87531 | 10.12469 |
| *S. canicula* | 101778 | 173967 | 58.5042 | 41.4958 |
| *G. cuvier* | 152277 | 173967 | 87.53212 | 12.46788 |
| *C. perezii* | 158600 | 173967 | 91.16672 | 8.833284 |
| *L. nasus* | 128020 | 173967 | 73.58867 | 26.41133 |
| *P. glauca* | 152884 | 173967 | 87.88103 | 12.11897 |
| *M. canis* | 147206 | 173967 | 84.6172 | 15.3828 |
| *C. leucas* | 148820 | 173967 | 85.54496 | 14.45504 |
| *R. terraenovae* | 152351 | 173967 | 87.57465 | 12.42535 |
| *C. acronotus* | 159616 | 173967 | 91.75073 | 8.249266 |
| *C. maximus* | 17920 | 173967 | 10.3008 | 89.6992 |
| *C. taurus* | 150116 | 173967 | 86.28993 | 13.71007 |
| *C. carcharias* | 124004 | 173967 | 71.28019 | 28.71981 |
| *N. brevirostris* | 146036 | 173967 | 83.94466 | 16.05534 |
| *I. oxyrinchus* | 156376 | 173967 | 89.88831 | 10.11169 |

Table S4: Genes found to be under selection by the PAML branch-site tests. Genes reported here are those which had were found to be under selection using both tcoffee and guidance alignment pipelines. *p*-values reported are the highest values of those corrected across each analysis of each gene using Benjamini-Hochberg. All gene names from blastx searches of coding regions against the SwissProt database, with e-value less than 1e-10

|  | Ortholog | Top SwissProt blastx hit | Bayes empirical Bayes sites > 0.9 | Likelihood Ratio Test | Adjusted p-value |
| --- | --- | --- | --- | --- | --- |
| Endothermic tuna | OG0000027_1_rr_1.inclade4.ortho1_rr | *TRI35* Tripartite motif-containing protein 35 | 0 | 6.131832 | 0.013276923 |
|  | OG0000036_1_rr_1.inclade3.ortho1_rr | *SEM3G* Semaphorin-3G | 1 | 5.404268 | 0.020373595 |
|  | OG0000148_1_rr_1.inclade1.ortho1_rr | *CO4A* Complement C4-*A* | 1 | 14.21874 | 0.000519501 |
|  | OG0000192_1_rr_1.inclade1.ortho1_rr | *MPSF* M-protein, striated muscle | 0 | 9.723888 | 0.003555777 |
|  | OG0000192_1_rr_1.inclade2.ortho1_rr | *MPSF* M-protein, striated muscle | 1 | 7.978374 | 0.004733944 |
|  | OG0000229_1_rr_1.inclade1.ortho1_rr | *VPP2* V-type proton ATPase 116 kDa subunit a isoform *2* | 1 | 5.17927 | 0.037796338 |
|  | OG0000257_1_rr_1.inclade4.ortho1_rr | *CATK* Cathepsin K | 1 | 7.097002 | 0.0077213 |
|  | OG0000275_1_rr_1.inclade2.ortho1_rr | *TEAD3* Transcriptional enhancer factor TEF-*5* | 4 | 33.76852 | 6.21E-09 |
|  | OG0000282_1_rr_1.inclade1.ortho1_rr | *LADD* Ladderlectin | 0 | 10.59577 | 0.001133464 |
|  | OG0000313_1_rr_1.inclade2.ortho1_rr | *FHL3* Four and a half LIM domains protein *3* | 1 | 7.098266 | 0.009534629 |
|  | OG0000316_1_rr_1.inclade3.ortho1_rr | *LRC8D* Volume-regulated anion channel subunit LRRC8D | 1 | 9.679988 | 0.001862858 |
|  | OG0000324_1_rr_1.inclade1.ortho1_rr | *DRA* Mamu class II histocompatibility antigen, DR alpha chain | 2 | 9.656842 | 0.001886476 |
|  | OG0000325_1_rr_1.inclade3.ortho1_rr | *DEN5B* DENN domain-containing protein *5B* | 0 | 4.214094 | 0.04008947 |
|  | OG0000325_1_rr_1.inclade4.ortho1_rr | *DEN5B* DENN domain-containing protein *5B* | 1 | 6.248064 | 0.01590533 |
|  | OG0000338_1_rr_1.inclade1.ortho1_rr | *C1S* Complement C1s subcomponent | 0 | 6.133146 | 0.013267059 |
|  | OG0000340_1_rr_1.inclade3.ortho1_rr | *MK14A* Mitogen-activated protein kinase *14A* | 10 | 58.54618 | 1.32E-11 |
|  | OG0000405_1_rr_1.inclade2.ortho1_rr | *MFAP4* Microfibril-associated glycoprotein *4* | 2 | 7.72439 | 0.005447976 |
|  | OG0000433_1_rr_1.inclade2.ortho1_rr |  | 0 | 5.738546 | 0.02700768 |
|  | OG0000461_1_rr_1.inclade3.ortho1_rr | *CSCL1* CSC1-like protein *1* | 1 | 4.535222 | 0.03320415 |
|  | OG0000479_1_rr_1.inclade1.ortho1_rr | *SVIL* Supervillin | 2 | 5.507314 | 0.024179958 |
|  | OG0000609_1_rr_1.inclade2.ortho1_rr | *DTNB* Dystrobrevin beta | 1 | 4.499098 | 0.033912738 |
|  | OG0000639_1_rr_1.inclade1.ortho1_rr | *UBP15* Ubiquitin carboxyl-terminal hydrolase *15* | 2 | 10.00729 | 0.001559216 |
|  | OG0000654_1_rr_1.inclade1.ortho1_rr | *NFL* Neurofilament light polypeptide | 1 | 8.087166 | 0.004457982 |
|  | OG0000690_1_rr_1.inclade1.ortho1_rr | *TGM2* Protein-glutamine gamma-glutamyltransferase *2* | 3 | 5.036162 | 0.024823429 |
|  | OG0000702_1_rr_1.inclade1.ortho1_rr | *P3H3* Prolyl 3-hydroxylase *3* | 0 | 6.187806 | 0.012863357 |
|  | OG0000814_1_rr_1.inclade2.ortho1_rr | *ACOT1* Acyl-coenzyme A thioesterase *1* | 3 | 10.236 | 0.001692616 |
|  | OG0000844_1_rr_1.inclade2.ortho1_rr | *IGFN1* Immunoglobulin-like and fibronectin type III domain-containing protein *1* | 3 | 22.72183 | 1.87E-06 |
|  | OG0000968_1_rr_1.inclade1.ortho1_rr | *I5P2* Type II inositol 1,4,5-trisphosphate 5-phosphatase | 1 | 4.314646 | 0.046014488 |
|  | OG0001083_1_rr_1.inclade1.ortho1_rr | *NID1* Nidogen-*1* | 0 | 6.044012 | 0.027029903 |
|  | OG0001130_1_rr_1.inclade1.ortho1_rr | *LBR* Lamin-B receptor | 1 | 7.03371 | 0.00800911 |
|  | OG0001154_1_rr_1.inclade1.ortho1_rr | *AATC* Aspartate aminotransferase, cytoplasmic | 3 | 8.20019 | 0.00423023 |
|  | OG0001188_1_rr_1.inclade1.ortho1_rr | *CATD* Cathepsin *D* | 0 | 4.0201 | 0.044961048 |
|  | OG0001238_1_rr_1.inclade1.ortho1_rr | *PKHA1* Pleckstrin homology domain-containing family A member *1* | 4 | 49.88673 | 1.63E-12 |
|  | OG0001348_1_rr_1.inclade1.ortho1_rr | *APBA1* Amyloid beta A4 precursor protein-binding family A member *1* | 0 | 8.43025 | 0.003797391 |
|  | OG0001410_1_rr_1.inclade1.ortho1_rr | *NAGAB* Alpha-N-acetylgalactosaminidase | 12 | 6.935698 | 0.008708211 |
|  | OG0001415_1_rr_1.inclade2.ortho1_rr | *PECA1* Platelet endothelial cell adhesion molecule | 3 | 10.47229 | 0.005939654 |
|  | OG0001423_1_rr_1.inclade1.ortho1_rr | *SNP23* Synaptosomal-associated protein *23* | 1 | 13.87549 | 0.000195329 |
|  | OG0001615_1_rr_1.inclade3.ortho1_rr | *LIPL* Lipoprotein lipase | 1 | 5.532782 | 0.030695 |
|  | OG0001673_1_rr_1.inclade1.ortho1_rr | *ACOT4* Acyl-coenzyme A thioesterase *4* | 1 | 5.232344 | 0.022170635 |
|  | OG0001715_1_rr_1.inclade1.ortho1_rr | *CO4A2* Collagen alpha-2(IV) chain | 0 | 16.62604 | 4.55E-05 |
|  | OG0001771_1_rr_1.inclade2.ortho1_rr | *CDYL2* Chromodomain Y-like protein *2* | 2 | 7.057804 | 0.013074747 |
|  | OG0001817_1_rr_1.inclade1.ortho1_rr | *GRN* Granulins | 0 | 5.126678 | 0.029978164 |
|  | OG0001858_1_rr_1.inclade2.ortho1_rr | *KDEL1* KDEL motif-containing protein *1* | 0 | 12.24956 | 0.00131757 |
|  | OG0002150_1_rr_1.inclade1.ortho1_rr | *LIFR* Leukemia inhibitory factor receptor | 0 | 5.72134 | 0.016759935 |
|  | OG0002166_1_rr_1.inclade2.ortho1_rr | *NPAL3* NIPA-like protein *3* | 1 | 7.338426 | 0.010816455 |
|  | OG0002246_1_rr_1.inclade1.ortho1_rr | *TPISB* Triosephosphate isomerase *B* | 1 | 10.56293 | 0.001176862 |
|  | OG0002291_1_rr_1.inclade2.ortho1_rr | *BZW1A* Basic leucine zipper and W2 domain-containing protein *1-A* | 1 | 10.97517 | 0.000923406 |
|  | OG0002367_1_rr_1.inclade1.ortho1_rr | *MALT1* Mucosa-associated lymphoid tissue lymphoma translocation protein 1 homolog | 0 | 7.317592 | 0.009862294 |
|  | OG0002408_1_rr_1.inclade1.ortho1_rr |  | 3 | 23.67878 | 0.002418631 |
|  | OG0002408_1_rr_1.inclade2.ortho1_rr |  | 3 | 13.75706 | 0.003767425 |
|  | OG0002469_1_rr_1.inclade1.ortho1_rr | *CLPB* Caseinolytic peptidase B protein homolog | 0 | 6.313584 | 0.012113354 |
|  | OG0002503_1_rr_1.inclade1.ortho1_rr | *FKBP4* Peptidyl-prolyl cis-trans isomerase FKBP4 | 0 | 7.610536 | 0.005956148 |
|  | OG0002530_1_rr_1.inclade1.ortho1_rr | *ZN410* Zinc finger protein 410 | 0 | 5.109128 | 0.023800911 |
|  | OG0002842_1_rr_1.inclade1.ortho1_rr | *PGPI* Pyroglutamyl-peptidase 1 | 0 | 5.233972 | 0.022386409 |
|  | OG0002847_1_rr_1.inclade2.ortho1_rr | *GRAM2* GRAM domain-containing protein 2 | 1 | 4.324488 | 0.040426745 |
|  | OG0002883_1_rr_1.inclade1.ortho1_rr | *TNF6B* Tumor necrosis factor receptor superfamily member *6B* | 1 | 6.553218 | 0.021771728 |
|  | OG0003168_1_rr_1.inclade1.ortho1_rr | *4F2* 4F2 cell-surface antigen heavy chain | 1 | 9.225924 | 0.002386122 |
|  | OG0003232_1_rr_1.inclade2.ortho1_rr | *I11RA* Interleukin-11 receptor subunit alpha | 3 | 6.432184 | 0.011207069 |
|  | OG0003236_1_rr_1.inclade1.ortho1_rr | *RIR1* Ribonucleoside-diphosphate reductase large subunit | 2 | 10.73361 | 0.001052071 |
|  | OG0003374_1_rr_1.inclade1.ortho1_rr | *ASUN* Protein asunder homolog | 0 | 7.456808 | 0.006532352 |
|  | OG0003443_1_rr_1.inclade1.ortho1_rr | *TCO2* Transcobalamin-*2* | 0 | 11.76838 | 0.000602455 |
|  | OG0003632_1_rr_1.inclade1.ortho1_rr | 5NTD 5'-nucleotidase | 4 | 15.67692 | 7.51E-05 |
|  | OG0003686_1_rr_1.inclade1.ortho1_rr | *MOT4* Monocarboxylate transporter *4* | 0 | 4.638676 | 0.037019205 |
|  | OG0003717_1_rr_1.inclade1.ortho1_rr | *LRC17* Leucine-rich repeat-containing protein *17* | 0 | 7.227424 | 0.007179816 |
|  | OG0003915_1_rr_1.inclade2.ortho1_rr | *DRAM1* DNA damage-regulated autophagy modulator protein *1* | 1 | 5.889014 | 0.015395576 |
|  | OG0004024_1_rr_1.inclade1.ortho1_rr | *ODO2* Dihydrolipoyllysine-residue succinyltransferase component of 2-oxoglutarate dehydrogenase complex, mitochondrial | 1 | 8.084154 | 0.004482842 |
|  | OG0004121_1_rr_1.inclade1.ortho1_rr | *SERPH* Serpin *H1* | 1 | 12.3458 | 0.00044198 |
|  | OG0004177_1_rr_1.inclade1.ortho1_rr | *ARRD1* Arrestin domain-containing protein *1* | 2 | 5.80303 | 0.049777974 |
|  | OG0004384_1_rr_1.inclade1.ortho1_rr | *TIGRA* Probable fructose-2,6-bisphosphatase | 1 | 7.771998 | 0.005306233 |
|  | OG0004399_1_rr_1.inclade1.ortho1_rr | *GMEB1* Glucocorticoid modulatory element-binding protein *1* | 1 | 7.818976 | 0.005170048 |
|  | OG0004410_1_rr_1.inclade1.ortho1_rr | *MTMRA* Myotubularin-related protein *10* | 0 | 4.422894 | 0.041497363 |
|  | OG0004635_1_rr_1.inclade2.ortho1_rr | *MYOZ2* Myozenin-*2* | 3 | 6.616368 | 0.010104569 |
|  | OG0004662_1_rr_1.inclade1.ortho1_rr | *NRAP* Nebulin-related-anchoring protein | 1 | 6.789046 | 0.009171891 |
|  | OG0004746_1_rr_1.inclade1.ortho1_rr | *MOGS* Mannosyl-oligosaccharide glucosidase | 1 | 6.735464 | 0.010163411 |
|  | OG0004753_1_rr_1.inclade1.ortho1_rr | *LPP60* 60 kDa lysophospholipase | 6 | 7.158704 | 0.022519294 |
|  | OG0004756_1_rr_1.inclade1.ortho1_rr | *ARY1* Arylamine N-acetyltransferase, pineal gland isozyme NAT-*10* | 0 | 6.573794 | 0.01034925 |
|  | OG0004849_1_rr_1.inclade1.ortho1_rr | *VISI* Visinin | 2 | 10.9746 | 0.000923693 |
|  | OG0004866_1_rr_1.inclade1.ortho1_rr | *MCAT* Mitochondrial carnitine/acylcarnitine carrier protein | 1 | 9.486002 | 0.004253039 |
|  | OG0004936_1_rr_1.inclade1.ortho1_rr | *TMCO4* Transmembrane and coiled-coil domain-containing protein *4* | 0 | 4.262972 | 0.038951718 |
|  | OG0005089_1_rr_1.inclade1.ortho1_rr | *HSPB1* Heat shock protein beta-*1* | 1 | 8.063888 | 0.004823971 |
|  | OG0005115_1_rr_1.inclade2.ortho1_rr | *HEMO* Hemopexin | 2 | 12.66629 | 0.004258313 |
|  | OG0005238_1_rr_1.inclade1.ortho1_rr | *PANK4* Pantothenate kinase *4* | 0 | 5.053338 | 0.024578544 |
|  | OG0005241_1_rr_1.inclade2.ortho1_rr | *DJC30* DnaJ homolog subfamily C member *30* | 0 | 7.2942 | 0.006917757 |
|  | OG0005313_1_rr_1.inclade2.ortho1_rr | *MBP* Myelin basic protein | 2 | 8.519988 | 0.00364558 |
|  | OG0005315_1_rr_1.inclade1.ortho1_rr | *RFESD* Rieske domain-containing protein | 3 | 5.565426 | 0.019337458 |
|  | OG0005467_1_rr_1.inclade1.ortho1_rr | *RHG18* Rho GTPase-activating protein *18* | 0 | 5.508506 | 0.019008863 |
|  | OG0005898_1_rr_1.inclade1.ortho1_rr | *RBP1* RalA-binding protein *1* | 1 | 5.791912 | 0.016618217 |
|  | OG0005945_1_rr_1.inclade1.ortho1_rr | *CF136* Uncharacterized protein C6orf136 homolog | 0 | 6.846412 | 0.023228468 |
|  | OG0006210_1_rr_1.inclade1.ortho1_rr | *PNBA* Para-nitrobenzyl esterase | 1 | 6.644802 | 0.009953711 |
|  | OG0006328_1_rr_1.inclade1.ortho1_rr | *WDR1* WD repeat-containing protein *1* | 1 | 5.937178 | 0.014824753 |
|  | OG0006362_1_rr_1.inclade1.ortho1_rr | *MPRD* Cation-dependent mannose-6-phosphate receptor | 1 | 10.92433 | 0.000949096 |
|  | OG0006378_1_rr_1.inclade1.ortho1_rr | *ARPC5* Actin-related protein 2/3 complex subunit *5* | 2 | 7.393844 | 0.006544747 |
|  | OG0006396_1_rr_1.inclade1.ortho1_rr | *NSUN5* Probable 28S rRNA (cytosine-C(5))-methyltransferase | 2 | 13.14841 | 0.00036116 |
|  | OG0006460_1_rr_1.inclade1.ortho1_rr | *CHP3* Calcineurin B homologous protein *3* | 3 | 6.444764 | 0.01119418 |
|  | OG0006580_1_rr_1.inclade1.ortho1_rr | *TCHP* Trichoplein keratin filament-binding protein | 1 | 7.671726 | 0.005674828 |
|  | OG0006612_1_rr_1.inclade1.ortho1_rr | *LYG* Lysozyme g | 0 | 4.4274 | 0.035366343 |
|  | OG0006656_1_rr_1.inclade1.ortho1_rr |  | 0 | 5.843858 | 0.016910604 |
|  | OG0006943_1_rr_1.inclade1.ortho1_rr | *EFTU* Elongation factor Tu, mitochondrial | 1 | 6.422976 | 0.011265335 |
|  | OG0006959_1_rr_1.inclade1.ortho1_rr | *IRF8* Interferon regulatory factor 8 | 2 | 5.897482 | 0.017705642 |
|  | OG0006971_1_rr_1.inclade1.ortho1_rr | *ADML* Adrenomedulin | 3 | 6.883216 | 0.008924399 |
|  | OG0007076_1_rr_1.inclade1.ortho1_rr | *TM55A* Type 2 phosphatidylinositol 4,5-bisphosphate 4-phosphatase | 1 | 9.53429 | 0.003600695 |
|  | OG0007092_1_rr_1.inclade1.ortho1_rr | *S22AG* Solute carrier family 22 member 16 | 1 | 8.015782 | 0.004637145 |
|  | OG0007093_1_rr_1.inclade1.ortho1_rr | *MOT13* Monocarboxylate transporter 13 | 0 | 7.06535 | 0.007858896 |
|  | OG0007164_1_rr_1.inclade1.ortho1_rr | *CD3Z* T-cell surface glycoprotein CD3 zeta chain | 1 | 6.133522 | 0.026062554 |
|  | OG0007417_1_rr_1.inclade1.ortho1_rr | *F210B* Protein FAM210B | 0 | 5.28305 | 0.033290791 |
|  | OG0007452_1_rr_1.inclade1.ortho1_rr |  | 0 | 4.787488 | 0.028667205 |
|  | OG0007486_1_rr_1.inclade1.ortho1_rr | *JGN1B* Protein jagunal homolog *1-B* | 1 | 7.562898 | 0.005958211 |
|  | OG0007626_1_rr_1.inclade1.ortho1_rr | *THIM* 3-ketoacyl-CoA thiolase, mitochondrial | 1 | 11.57164 | 0.000681116 |
|  | OG0007813_1_rr_1.inclade1.ortho1_rr | *ODB2* Lipoamide acyltransferase component of branched-chain alpha-keto acid dehydrogenase complex, mitochondrial | 1 | 8.470034 | 0.003909694 |
|  | OG0007862_1_rr_1.inclade1.ortho1_rr | *SCF* Kit ligand | 1 | 8.158176 | 0.004286746 |
|  | OG0007868_1_rr_1.inclade1.ortho1_rr | *APT* Adenine phosphoribosyltransferase | 1 | 4.762144 | 0.029092288 |
|  | OG0007903_1_rr_1.inclade1.ortho1_rr | *GOGA5* Golgin subfamily A member *5* | 0 | 5.986916 | 0.014412379 |
|  | OG0008012_1_rr_1.inclade1.ortho1_rr | *TWF2* Twinfilin-*2* | 1 | 8.621832 | 0.003717708 |
|  | OG0008069_1_rr_1.inclade1.ortho1_rr | *TM9S3* Transmembrane 9 superfamily member *3* | 0 | 8.585938 | 0.01881658 |
|  | OG0008244_1_rr_1.inclade1.ortho1_rr | *IRA1A* Interferon alpha/beta receptor *1a* | 1 | 11.37307 | 0.000767125 |
|  | OG0008297_1_rr_1.inclade1.ortho1_rr | *CKAP4* Cytoskeleton-associated protein *4* | 0 | 8.795646 | 0.003943855 |
|  | OG0008373_1_rr_1.inclade1.ortho1_rr | *LMOD3* Leiomodin-*3* | 1 | 5.879246 | 0.017690597 |
|  | OG0008407_1_rr_1.inclade1.ortho1_rr | *KBL* 2-amino-3-ketobutyrate coenzyme A ligase, mitochondrial | 1 | 3.853286 | 0.0496812 |
|  | OG0008554_1_rr_1.inclade1.ortho1_rr | *SODC* Superoxide dismutase [Cu-Zn] | 2 | 4.040684 | 0.044416448 |
|  | OG0008583_1_rr_1.inclade1.ortho1_rr | *TXLNB* Beta-taxilin | 1 | 7.834606 | 0.005125532 |
|  | OG0008607_1_rr_1.inclade1.ortho1_rr | *RASLC* Ras-like protein family member *12* | 0 | 6.478962 | 0.010915871 |
|  | OG0008659_1_rr_1.inclade1.ortho1_rr | *PSMD9* 26S proteasome non-ATPase regulatory subunit *9* | 1 | 7.411884 | 0.006479444 |
|  | OG0008779_1_rr_1.inclade1.ortho1_rr | *ENKD1* Enkurin domain-containing protein *1* | 0 | 4.422828 | 0.035461218 |
|  | OG0008817_1_rr_1.unrooted-ortho_rr | *RYR1* Ryanodine receptor *1* | 1 | 4.286042 | 0.038426522 |
|  | OG0008981_1_rr_1.inclade1.ortho1_rr | *COPT1* High affinity copper uptake protein *1* | 0 | 6.0431 | 0.013960749 |
|  | OG0009064_1_rr_1.inclade1.ortho1_rr | *NLRC3* Protein NLRC*3* | 0 | 7.276028 | 0.007236274 |
|  | OG0009451_1_rr_1.inclade1.ortho1_rr | *PBDC1* Protein PBDC*1* | 1 | 9.182442 | 0.002443478 |
|  | OG0009487_1_rr_1.inclade1.ortho1_rr | *GPR1* G-protein coupled receptor *1* | 1 | 8.581806 | 0.003694207 |
|  | OG0009515_1_rr_1.inclade1.ortho1_rr | *PMGE* Bisphosphoglycerate mutase | 1 | 5.319914 | 0.021468502 |
|  | OG0009526_1_rr_1.inclade1.ortho1_rr | *TIFA* TRAF-interacting protein with FHA domain-containing protein *A* | 1 | 8.912028 | 0.002889177 |
|  | OG0009909_1_rr_1.inclade1.ortho1_rr | *RN214* RING finger protein *214* | 0 | 5.902152 | 0.015122397 |
|  | OG0009948_1_rr_1.inclade1.ortho1_rr | *PEF1* Peflin | 3 | 10.69641 | 0.001174949 |
|  | OG0009963_1_rr_1.inclade1.ortho1_rr | *HSPB8* Heat shock protein beta-*8* | 1 | 9.059336 | 0.00261358 |
|  | OG0010293_1_rr_1.inclade1.ortho1_rr | *LRRC2* Leucine-rich repeat-containing protein *2* | 1 | 6.159072 | 0.013073978 |
|  | OG0010642_1_rr_1.inclade1.ortho1_rr | *ACOT13* Acyl-coenzyme A thioesterase *13* | 0 | 9.343788 | 0.002278824 |
|  | OG0010698_1_rr_1.inclade1.ortho1_rr |  | 3 | 8.91754 | 0.002824447 |
|  | OG0011412_1_rr_1.inclade1.ortho1_rr |  | 0 | 6.07433 | 0.013716032 |
|  | OG0011418_1_rr_1.inclade1.ortho1_rr | *MYSM1* Histone H2A deubiquitinase MYSM*1* | 0 | 7.28415 | 0.006956564 |
|  | OG0013339_1_rr_1.unrooted-ortho_rr | *GLYG* Glycogenin-*1* | 2 | 6.893528 | 0.008744195 |
| Endothermic sharks | OG0000096_1_rr_1.inclade2.ortho1_rr | *ST2B1* Sulfotransferase family cytosolic 2B member *1* | 0 | 4.385486 | 0.037085347 |
|  | OG0000173_1_rr_1.inclade1.ortho1_rr | *ILEU* Leukocyte elastase inhibitor | 1 | 10.53614 | 0.001170628 |
|  | OG0000173_1_rr_1.inclade2.ortho1_rr | *ILEU* Leukocyte elastase inhibitor | 0 | 5.910922 | 0.01511311 |
|  | OG0000606_1_rr_1.inclade1.ortho1_rr | *PRDX3* Thioredoxin-dependent peroxide reductase, mitochondrial | 1 | 5.473002 | 0.019312425 |
|  | OG0001373_1_rr_1.inclade2.ortho1_rr | *DNSL3* Deoxyribonuclease gamma | 6 | 6.071544 | 0.013737682 |
|  | OG0001540_1_rr_1.inclade1.ortho1_rr | *GLYG* Glycogenin-*1* | 2 | 5.188774 | 0.022733248 |
|  | OG0001669_1_rr_1.inclade1.ortho1_rr | *SAT1* Diamine acetyltransferase *1* | 1 | 5.90952 | 0.020420387 |
|  | OG0001852_1_rr_1.inclade2.ortho1_rr | *COX41* Cytochrome c oxidase subunit *4* isoform *1*, mitochondrial | 0 | 6.11228 | 0.013424584 |
|  | OG0001929_1_rr_1.inclade2.ortho1_rr | *CXAR* Coxsackievirus and adenovirus receptor homolog | 1 | 6.653136 | 0.011076229 |
|  | OG0001950_1_rr_1.inclade1.ortho1_rr | *MTX1* Metaxin-*1* | 2 | 22.78031 | 1.15E-05 |
|  | OG0002657_1_rr_1.inclade1.ortho1_rr | *NPC2* Epididymal secretory protein E1 | 1 | 4.027014 | 0.044777127 |
|  | OG0003057_1_rr_1.inclade1.ortho1_rr | *TPC6B* Trafficking protein particle complex subunit 6B | 1 | 7.68435 | 0.005571495 |
|  | OG0004163_1_rr_1.inclade1.ortho1_rr | *TM2D3* TM*2* domain-containing protein *3* | 1 | 5.001254 | 0.027224738 |
|  | OG0005956_1_rr_1.inclade1.ortho1_rr | *MYG* Myoglobin | 1 | 7.27536 | 0.006990688 |
|  | OG0006034_1_rr_1.inclade1.ortho1_rr | *SAHHA* Adenosylhomocysteinase *A* | 1 | 12.28923 | 0.000457027 |
|  | OG0007128_1_rr_1.inclade1.ortho1_rr |  | 7 | 9.33698 | 0.003279245 |
|  | OG0007201_1_rr_1.inclade1.ortho1_rr | *BTNL2* Butyrophilin-like protein *2* | 1 | 6.189978 | 0.012847578 |
|  | OG0009761_1_rr_1.inclade1.ortho1_rr |  | 0 | 6.329692 | 0.019239683 |
|  | OG0009893_1_rr_1.inclade1.ortho1_rr | *RIAD1* RIIa domain-containing protein *1* | 0 | 10.29881 | 0.001499764 |
